# Molecular analysis of the *APC *and *MUTYH *genes in Galician and Catalonian FAP families: a different spectrum of mutations?

**DOI:** 10.1186/1471-2350-10-57

**Published:** 2009-06-16

**Authors:** Nuria Gómez-Fernández, Sergi Castellví-Bel, Ceres Fernández-Rozadilla, Francesc Balaguer, Jenifer Muñoz, Irene Madrigal, Montserrat Milà, Begoña Graña, Ana Vega, Antoni Castells, Ángel Carracedo, Clara Ruiz-Ponte

**Affiliations:** 1 Fundación Pública Galega de Medicina Xenómica (FPGMX)-SERGAS, Grupo de Medicina Xenómica (GMX)-USC, CIBERER, Santiago de Compostela, Galicia, Spain; 2 Departmento de Gastroenterología, Institut de Malalties Digestives i Metabòliques, Hospital Clínic, CIBEREHD, IDIBAPS, Barcelona, Catalonia, Spain; 3 CIBER-ER, Departamento de Bioquímica y Genética Molecular, Hospital Clínic, IDIBAPS, Barcelona, Catalonia, Spain; 4 Servicio de Oncoloxía Médica, Hospital Clínico-CHUS. Santiago de Compostela, Galicia, Spain

## Abstract

**Background:**

Familial adenomatous polyposis (FAP) is an autosomal dominant-inherited colorectal cancer syndrome, caused by germline mutations in the *APC *gene. Recently, biallelic mutations in *MUTYH *have also been identified in patients with multiple colorectal adenomas and in *APC*-negative patients with FAP. The aim of this work is therefore to determine the frequency of *APC *and *MUTYH *mutations among FAP families from two Spanish populations.

**Methods:**

Eighty-two unrelated patients with classical or attenuated FAP were screened for *APC *germline mutations. *MUTYH *analysis was then conducted in those *APC*-negative families and in 9 additional patients from a previous study. Direct sequencing, SSCP analysis and TaqMan genotyping were used to identify point and frameshift mutations, meanwhile large rearrangements in the *APC *gene were screened by multiplex ligation-dependent probe amplification (MLPA).

**Results:**

*APC *germline mutations were found in 39% of the patients and, despite the great number of genetic variants described so far in this gene, seven new mutations were identified. The two hotspots at codons 1061 and 1309 of the *APC *gene accounted for 9,4% of the *APC*-positive families, although they were underrepresented in Galician samples. The deletion at codon 1061 was not found in 19 *APC*-positive Galician patients but represented 23% of the Catalonian positive families (p = 0,058). The same trend was observed at codon 1309, even though statistical analysis showed no significance between populations. Twenty-four percent of the *APC*-negative patients carried biallelic *MUTYH *germline mutations, and showed an attenuated polyposis phenotype generally without extracolonic manifestations. New genetic variants were found, as well as the two hotspots already reported (p.Tyr165Cys and p.Gly382Asp).

**Conclusion:**

The results we present indicate that in Galician patients the frequency of the hotspot at codon 1061 in *APC *differs significantly from the Catalonian and also other Caucasian populations. Similar results had already been obtained in a previous study and could be due to the genetic isolation of the Galician population. *MUTYH *analysis is also recommended for all *APC*-negative families, even if a recessive inheritance is not confirmed.

## Background

Familial adenomatous polyposis (FAP; OMIM#175100) is a rare autosomal dominant colorectal cancer predisposition syndrome, characterised by the presence of hundreds to thousands of adenomatous polyps in the colon and rectum from an early age. In the absence of prophylactic surgery, colorectal cancer (CRC) is the inevitable consequence of FAP. Extracolonic manifestations such as osteomas, congenital hypertrophy of the retinal pigment epithelium (CHRPE), desmoid tumors, sebaceous cysts, hepatoblastoma, upper gastrointestinal tumors or thyroid carcinoma are also associated with FAP [1]. Attenuated FAP (AFAP) is a clinical variant characterised by the presence of fewer than 100 colonic polyps, and often has a later age of onset of polyposis and CRC [2].

The genetic basis of most cases of FAP is a germline mutation of the adenomatous polyposis coli (*APC*) gene (5q21), which encodes a tumor suppressor protein involved in regulation of cell proliferation and chromosome segregation [3]. About 90% of the germline mutations in FAP result in truncation of the APC protein and are mainly located within exon 15 [4]. In AFAP, germline mutations have been generally detected either in exon 9 or the 5' and 3' ends of the gene [5].

It is known that *APC *germline mutations are not present in approximately 10–30% of FAP patients and in up to 90% of AFAP patients [6]. Recently, patients with multiple colorectal adenomas and also patients with FAP but without detectable germline *APC *mutations have been found to carry biallelic mutations in the base-excision-repair gene *MUTYH (MYH) *[7]. This base excision repair (BER) pathway is necessary to repair DNA damage caused by reactive oxygen species. The DNA glycosylase MUTYH removes adenines from mispairs with 8-oxoguanine that occur during the replication of oxidized DNA. Failure to correct these mispairs consequently leads to G:C→T:A tranversion mutations in tumors that resulted in the discovery of *MUTYH*-associated polyposis (MAP), which shows an autosomal recessive inheritance pattern [8]. Two mutational hotspots have been so far identified in the *MUTYH *gene: p.Tyr165Cys and p.Gly382Asp, accounting for approximately 78% of the mutations identified in affected Caucasians [9].

In this study, we examined the mutational spectrum of the *APC *gene in patients with polyposis from two Spanish populations, and also the contribution of *MUTYH *germline mutations in those *APC*-negative patients.

## Methods

### Patients and DNA isolation

The sample studied consisted of 82 unrelated cases with FAP (>100 colorectal adenomas) or AFAP (5–100 colorectal adenomas). All the patients were included in the study based on colonoscopic findings and/or positive family history. Forty-eight samples were submitted for mutation analysis at the Galician Public Foundation of Genomic Medicine (FPGMX) from health centers across Galicia, and 34 were attended in the at-risk clinic for CRC of the Hospital Clinic in Barcelona. Written informed consent was obtained for each patient before mutation analysis, according to the protocols approved by the ethics review boards of the Hospitals and in compliance with the Helsinki declaration.

All patients were screened for *APC *germline mutations, and when negative, *MUTYH *was analysed. *MUTYH *was also studied in 9 *APC-*negative families included in a previous article [10].

Clinical features for patients with detected mutations, including age of onset, number of adenomas, colorectal cancer diagnosis, extracolonic diseases and family history, if present, are listed in Tables 1 (*APC*) [5,10-20] and 2 (*MUTYH*) [7,8,21,22].

**Table 1:** Phenotypic features and germline mutations identified in *APC*-positive patients.

Patient ID	Onset age	Number of Adenomas	CRC	ED	Family history (age at diagnosis)	Mutation	Exon	Ref
GAL-27	42	>100	No	No	Father: CRC(36) Grandmother: CRC(60)	c.(?_30)_(*220_?)*del*	Whole allele	[11]

GAL-16	23	100	No	DT OST	NA	c.(?_30)_(*220_?)*del*	Whole allele	[11]

GAL-14	20	>100	No	?	No	c. 1-?_8532+?*del*	1 to 15	[12]

GAL-07	50	15	No	No	Affected mother (?)	**c.147-150*del*ACAA (p.Lys49Asn*fs*X20)**	2	This study

GAL-15	33	>100	No	?	No	c.423-?_531+?*del*	4	[5]

GAL-11	41	>100	Yes	No	Father: CRC (?)	c.646C>T (p.Arg216X)	6	[13]

GAL-26	15	>100	No	No	Father: FAP+CRC (47)	c.646 C>T (p.Arg216X)	6	[13]

GAL-09	33	12	No	No	No	c.994C>T (p.Arg332X)	9	[5]

GAL-10	64	50	Yes	No	Mother: CRC (68) Aunt: CRC (60)	c.1072C>T (p.Gln358X)	9	[14]

GAL-19	NA	20–50	No	No	Mother: CRC (46)	**c.1402 G>T (p.Glu468X)**	10	This study

GAL-13	20	>100	Yes	No	No	c.1620_1621*dup*A (p.Gln541Thr*fs*X19)	12	[15]

GAL-18	33	>50	No	No	Father: CRC (42)	c.1682*dup*A (p.Thr562Asn*fs*X19)	13	[10]

GAL-02	30	0	No	PC	No	c.1756 A>T (p.Lys586X)	14	[16]

GAL-01	23	<100	No	No	Brother: FAP (20s)	c.2413C>T (p.Arg805X)	15	[17]

GAL-17	41	100	Yes	OST	Father CRC (45), Sister CRC (34)	**c.2900*del*T (p.Val967Ala*fs*X13)**	15	This study

GAL-12	34	<100	No	No	Father: CRC (40s) Uncle: CRC (40s) Grandfather: CRC(40s)	c.3467_3470*del*AAGA (p.Glu1156Gly*fs*X8)	15	[18]

GAL-24	52	>100	No	CHRPE	No	c.3927_3931*del*AAAGA (p.Glu1309Asp*fs*X4)	15	[18]

GAL-04	60	>100	Yes	Others	No	c.4033G>T (p.Glu1345X)	15	[19]

GAL-25	43	>100	Yes	No	NA	**c.4219-4220*del*AG p.Ser1407X*fs*X1**	15	This study

CAT-12	21	>100	No	No	Mother: FAP+ CRC (49) Sister: FAP (31)	c.(?_30)_(*220_?)*del*	Whole allele	[11]

CAT-13	20	20–50	No	No	Father: FAP (?) Brother: FAP (?)	c.423-?_531+?*del*	4	[5]

CAT-01	46	100	Yes	No	Brother: CRC+FAP(53) Brother: CRC+FAP(59)	c.994C>T (p.Arg332X)	9	[5]

CAT-02	32	>100	No	DP	No	**c.2934_2935*del*AA (p.Gln978His*fs*X6)**	15	This study

CAT-03	38	40–60	No	DT	Father: CRC (46) Brother: FAP	c.3183_3187*del*ACAAA (p.Lys1061Lys*fs*X2)	15	[18]

CAT-04	20	>100	No	No	Mother: FAP + CRC (?) Cousin: CRC (52)	c.3183_3187*del*ACAAA (p.Lys1061Lys*fs*X2)	15	[18]

CAT-05	39	>100	No	DT	Sister: FAP (41)	c.3183_3187*del*ACAAA (p.Lys1061Lys*fs*X2)	15	[18]

CAT-06	38	>100	Yes	No	Brother: FAP Father: FAP + CRC (?)	**c.3329C>A (p.Ser1110X)**	15	This study

CAT-07	NA	NA	NA	NA	NA	**c.3329C>A (p.Ser1110X)**	15	This study

CAT-08	27	>100	No	No	Brother: FAP (29) Father: FAP (41) Grandmother: FAP(30)	**c.3531*del*T (p.Ile1177Met*fs*X5)**	15	This study

CAT-09	NA	NA	NA	DP	NA	c.3631_3632*del*AT (p.Met1211Val*fs*X5)	15	[20]

CAT-10	17	>100	No	FGP	Father: FAP (39) Uncle: FAP + CRC (?)	c.3927_3931*del*AAAGA (p.Glu1309Asp*fs*X4)	15	[18]

CAT-11	32	>100	No	FGP	Father: FAP (39) Uncle FAP + CRC(63). Aunt: FAP+ CRC(55) Grandmother: FAP+CRC(39)	c.3927_3931*del*AAAGA (p.Glu1309Asp*fs*X4)	15	[18]

ED: extracolonic disease; DT: desmoid tumor; OST: osteomas; NA: not available; PC: papillary carcinoma; CHRPE: congenital hypertrophy of the retinal pigmented epitelium; Others: Ovarian tumor and suprarenal adenoma; DP: duodenal polyps; FGP: fundic gland polyps.

**Table 2:** Phenotypic characteristics and germlime mutations identified in biallelic *MUTYH *carriers.

Patient ID	Onset age	Number of adenomas	CRC	ED	Family History (age at diagnosis)	*MUTYH*		Ref.
							
						1^st ^Mutation	2^nd ^mutation	
GAL-08	43	25–30	Yes	No	No	c.494A>G (p.Tyr165Cys)	c.494A>G (p.Tyr165Cys)	[7]

GAL-21	52	<100	No	No	No	c.494A>G (p.Tyr165Cys)	c.1145 G>A (p.Gly382Asp)	[7]

GAL-22	NA	40–60	No	No	Two siblings and mother: CRC (50s)	c.494A>G (p.Tyr165Cys)	c.1145 G>A (p.Gly382Asp)	[7]

GAL-05	58	<100	Yes	No	NA	c.494A>G (p.Tyr165Cys)	c.1145 G>A (p.Gly382Asp)	[7]

GAL-06	NA	40–100	No	No	Sister: AFAP (?)	c.494A>G (p.Tyr165Cys)	c.1145 G>A (p.Gly382Asp)	[7]

GAL-20	45	<100	No	No	Two siblings: AFAP+CRC (?)	c.1131 C>T (p.Gln377X)	c.1145 G>A (p.Gly382Asp)	[7,21]

GAL-03	44	31–100	Yes	No	No	c.1145 G>A (p.Gly382Asp)	c.1145 G>A (p.Gly382Asp)	[7]

GAL-23	62	>30	Yes	No	No	c.1186_1187*ins*GG p.Glu396Gly*fs*X43	c.1186_1187*ins*GG p.Glu396Gly*fs*X43	[22]

CAT-15	44	5	Yes	No	Mother: BC(66) Brother:2 CRC (46)	c.494A>G (p.Tyr165Cys)	c.1103*del*C (p.Ala369Ala*fs*X26)	[7,8]

CAT-14	38	15–30	No	No	Father: CRC (?)	c.494A>G (p.Tyr165Cys)	c.1145 G>A (p.Gly382Asp)	[7]

CAT-17	60	>20	Yes	No	NA	c.1145G>A (p.Gly382Asp)	c.1145G>A (p.Gly382Asp)	[7]

CAT-16	45	40–50	No	No	Father: CRC (40)	c.1145G>A (p.Gly382Asp)	c.1145G>A (p.Gly382Asp)	[7]

CAT-18	45	70	Yes	No	No	c.1186_1187*ins*GG (p.Glu396Gly*fs*X43)	c.1186_1187*ins*GG (p.Glu396Gly*fs*X43)	[22]

CAT-19	69	0	Yes	BC (59)	Cousin: CRC (40)	c.1186_1187*ins*GG p.Glu396Gly*fs*X43	c.1186_1187*ins*GG p.Glu396Gly*fs*X43	[22]

ED: Extracolonic disease; BC: Breast cancer; NA: not available.

To allow comparison of our results, we used the *MUTYH *sequence used by previous authors (GenBank accession number: U63329) instead of the actual reference sequence (GenBank accession number: NM_012222), which has 11 additional codons in exon 3.

Genomic DNA from Galician and Catalonian samples was obtained from peripheral blood using the Wizard DNA extraction kit (Promega, Madison, WI), and the QIAamp DNA Blood Mini Kits (Qiagen, Hilden, Germany) respectively. Protocols were performed according to the manufacturer's instructions.

### Analysis of the APC gene

#### Sequence variants

Exonic and intronic splice-site defining regions were amplified for the *APC *gene. PCR conditions for exon 15 had already been described [10], whereas for exons 1–14, new primers were designed using the *Primer3 *software [23] in order to cover larger intronic regions [see Additional file 1].

Galician samples were analysed by direct DNA sequencing at the FPGMX. For the Catalonian samples, single strand conformational polymorphism (SSCP) analysis was performed at Hospital Clinic as an initial screening, as described [24]. Amplification products larger than 350 bp were previously digested with a suitable restriction enzyme. Any fragment showing a mobility shift was sequenced in order to identify the variant. Sequencing was performed in forward and reverse orientations using the BigDye terminator v.3.1. cycle sequencing kit (Applied Biosystems, Foster City, CA).

#### Genomic rearrangements

Large genomic rearrangements of the *APC *gene were evaluated with the *APC *multiplex ligation-dependent probe amplification (MLPA) kit [25], and performed according to the supplied protocol (SALSA MLPA KIT P043 APC, MRC-Holland, Amsterdam, The Netherlands). The amplicons were analysed in an ABI 3730 sequencer using GeneMapper v3.7 software (Applied Biosystems, Foster City, CA, USA). Peak heights of each fragment were compared to those of a control sample, and deletions or duplications were suspected when peak height differed by over 30%. Control DNA samples with known genomic rearrangements in *APC *were included in each batch of experiments. Positive results of large rearrangements were repeated in an independent assay and subsequently confirmed by other methods (FISH, cDNA analysis).

#### APC FISH

Fluorescent *in situ *hybridization (FISH) analysis was performed with RP11-3B10 and RP11-619D06 clones mapping in 5q21-q22. BAC clones were purchased from the BAC/PAC Resources of the Children's Hospital Oakland Research Institute (CHORI, Oakland, CA). Equal amounts of BAC DNA (200 ng) were labelled with Spectrum Orange (RP11-3B10) and Spectrum Green (RP11-619D06) by a standard nick-translation (Vysis, Downers Grove, IL, USA). This dual color probe was used to hybridize preparations of fixed cell nuclei and metaphases. Slides were visualized under an epi?uorescence microscope (Leica DMRXA). Images were captured by using a COHU camera and analysed with the Cytovision Ultra Workstation (Applied Imaging, Sunderland, UK).

#### RT-PCR

mRNA was isolated from blood using RNeasy^® ^Mini kit (Qiagen, Hilden, Germany). Synthesis of complementary DNA (cDNA) was performed with SuperScript™ II Reverse Transcriptase (Invitrogen, Carlsbad, USA). cDNA was then amplified using primers located in the adjacent exons to those regions potentially deleted. A positive cDNA control was included in every PCR. Amplification products were sequenced in an ABI3730 analyser.

### Analysis of the *MUTYH *gene

For every patient without detectable pathogenic mutations in *APC, *all *MUTYH *exons and their adjacent intronic splice sites were amplified using primers designed with the Primer3 software [23] [see Additional file 2].

Galician samples were analysed at the FPGMX center by sequencing each amplification fragment, as described above for the *APC *gene. Real-time PCR using Taqman probes, and SSCP analysis were performed for Catalonian samples at the Hospital Clinic. TaqMan genotyping included the analysis of the two most common mutations found to date in the *MUTYH *gene: p.Tyr165Cys and p.Gly382Asp, as well as the two rare mutations c.1103*del*C and c.1186_1187*ins*GG identified in our previous study [26]. This technique is based on allelic discrimination using allele-specific probes resolved on a 7300 Real Time PCR System (Applied Biosystems, Foster City, CA).

### Mutation nomenclature

All mutations were described following the guidelines proposed by the Human Genome Sequence Variation (HGSV) site and were referred to the cDNA sequences of *APC *(NM_000038) and *MUTYH *(U63329). Furthermore, all mutations were confirmed in two independent DNA extractions.

### Variants of Unknown Significance (VUS)

We examined 500 chromosomes from control individuals with no personal or family history of colorectal cancer, in order to estimate the frequency of VUS. Analysis was carried out by direct DNA sequencing (see above).

Polyphen software was used to test the potential role of missense variants. This prediction program is based on observed substitutions of the residues in homologous proteins [27].

### Statistical analyses

Ji-squared statistics with Fisher's correction were used to test for differences in *APC *and *MUTYH *mutation frequencies between the Galician and the Catalonian populations. Comparisons were also made for mutation frequencies at codons 1061 and 1309 of the *APC *gene. All statistics were estimated with the SPSS statistical software package (SPSS Inc., Chicago IL).

## Results and Discussion

### *APC *mutations

In this study, germline mutations in *APC *were found in 39% (32 out of 82) of the Spanish patients with FAP. Frameshift and nonsense mutations were the most frequently identified, and despite the great number of genetic variants described to date in the *APC *gene, seven new pathogenic mutations and two new VUS were reported. Clinical features displayed by *APC*-positive patients are shown on Table 1.

Five new frameshift mutations were identified: c.147_150*del*ACAA (p.Lys49Asn*fs*X20),c.2900*del*T(p.Val967Ala*fs*X13),c.2934_2935*del*AA(p.Gln978His*fs*X6),c.3531*del*T(Ile1177Met*fs*X5) and c.4219_4220*del*AG (p.Ser1407X*fs*X1) in patients GAL-07, GAL-17, CAT-02, CAT-08 and GAL-25, respectively. All of these mutations were deletions of few nucleotides, that give rise to premature stop codons (X) which would lead to truncated APC proteins. We also identified two additional nonsense mutations that generate premature stop codons: c.1402 G>T (p.Glu468X) in patient GAL-19, and c.3329C>A (p.Ser1110X) in two unrelated patients (CAT-06 and CAT-07).

The two new VUS: c.3165A>G (p.Ile1055Met) and c.5357G>C (p.Arg1786Thr), were found in GAL-47 and GAL-35. Both patients displayed an attenuated FAP phenotype with an onset at around forty. These variants were not detected in 500 chromosomes from a healthy control population. However, their absence from the control group cannot be taken as prove of a deleterious effect. *In silico *studies using Polyphen revealed the p.Arg1786Thr as "possibly damaging", while the p.Ile1055Met was reported as "benign". In these families it was not possible to study the co-segregation with the disease, so further functional studies are necessary to consider them deleterious.

**Figure 1:**
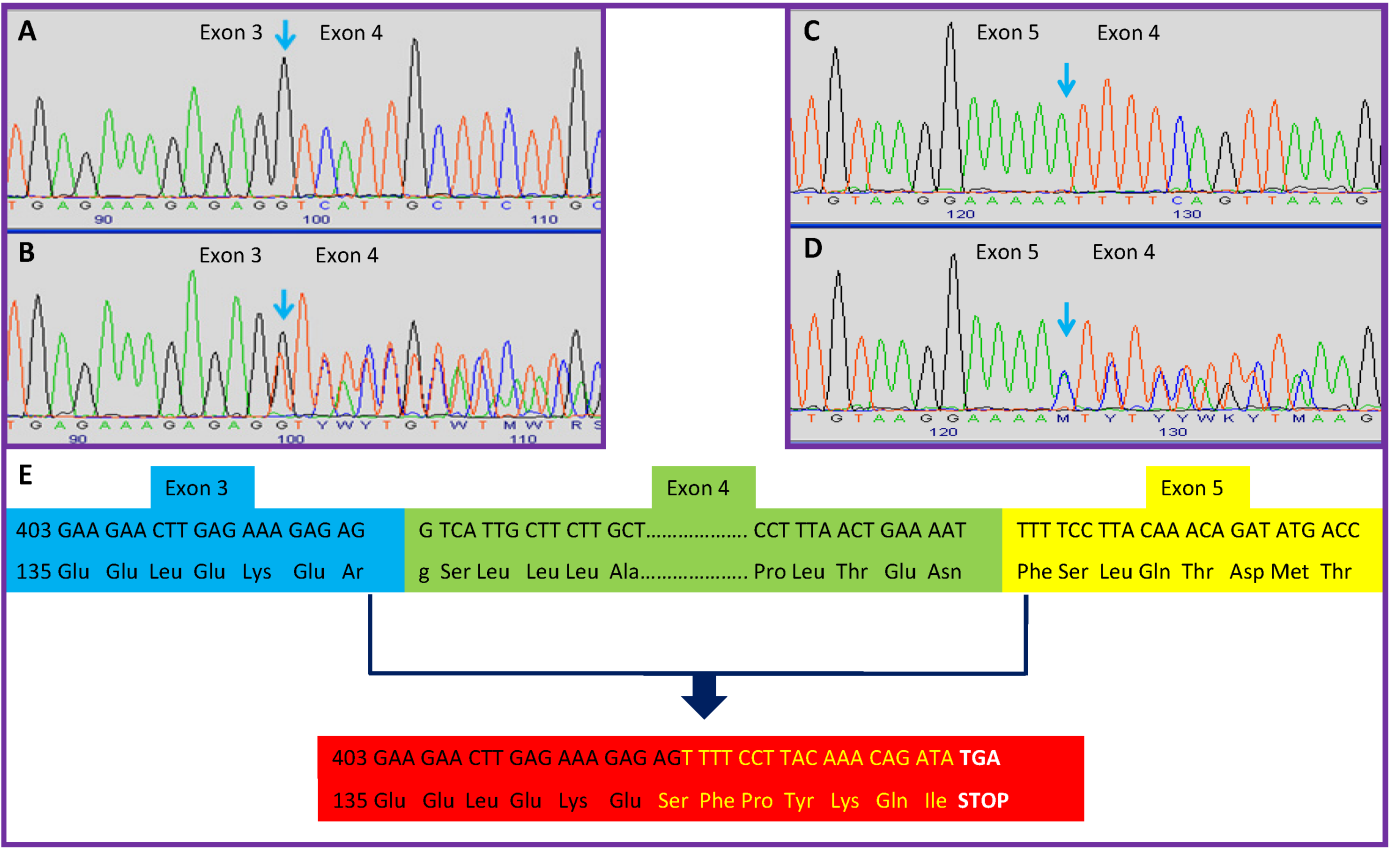
**cDNA analysis of the *APC *exon 4 deletion confirming the results obtained by MLPA**. A. forward reference sequence; B. forward sequence with exon 4 deletion; C. reverse reference sequence; D. reverse sequence with exon 4 deletion; E. Nucleotidic and aminoacidic sequences showing the effect of the exon 4 deletion, which results in a frameshift that creates a stop codon at residue 442.

**Figure 2:**
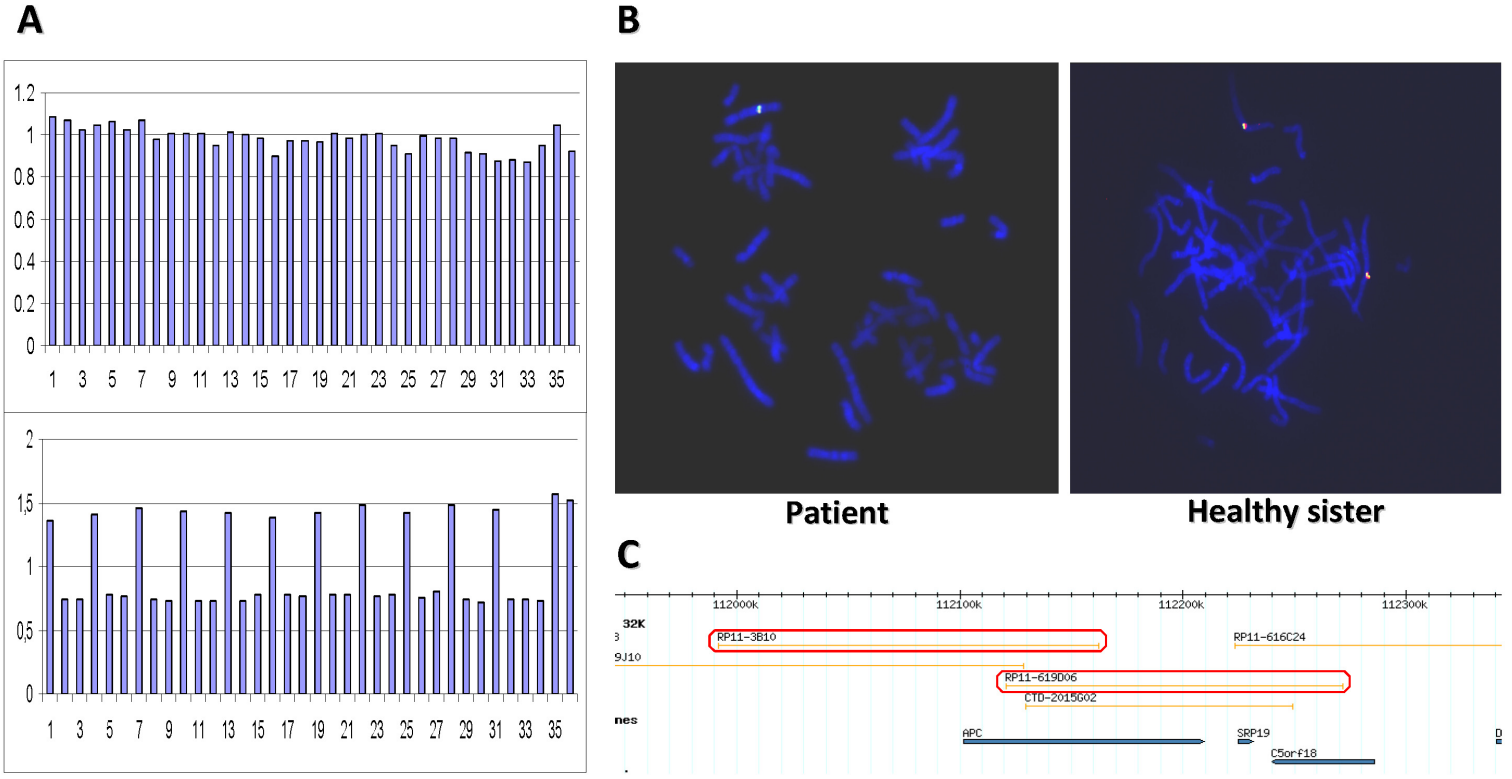
**Whole *APC *gene deletion detected by MLPA and FISH analysis**. A. Electropherograms of MLPA products showing a normal control and a deletion of the whole *APC *gene. B. FISH studies on metaphase spreads with clones RP11-3B10 (red probe) and RP11-619D06 (green probe) that map within the deletion, on the patient and a healthy sister. C. Physical mapping position, according to the hg17 assembly of the UCSC  of clones mapping the 5q21-22 region.

Large genomic deletions were found in 11% (6/56) of the families that tested *APC *mutation-negative by conventional techniques. This frequency is consistent with published data that comprise a range between 8–12% for such rearrangements [11,12,28]. Three different deletions were detected in 6 unrelated families: two exon 4 deletions (CAT-13 and GAL-15), an exon 1–15 deletion (GAL-14), and 3 whole-gene deletions (including the promoter) (CAT-12, GAL-27 and GAL-16). All of them were further confirmed by either cDNA studies (Figure 1) or FISH analysis (Figure 2).

Carriers of whole allelic deletions generally displayed a severe polyposis phenotype with an early onset of symptoms, as previously described [11]. A correlation between site of mutation and clinical phenotype was also observed for six of the seven new mutations identified. Mutations occurring at the beginning and middle of exon 15 were generally associated with a more severe phenotype than those located at the 5' and 3' ends of the gene, which is consistent with other studies [29]. Although mutations in exon 10 would then be associated with FAP, p.Glu468X was found in a patient classified as AFAP based on number of polyps (GAL-19) (Table 1).

Phenotypic differences about number of adenomas and extracolonic disease were observed in unrelated probands carrying the p.Lys1061LysfsX2 mutation (CAT-03 and CAT-04) and the whole gene deletions (CAT-12 and GAL-16) (Table 1). This phenotypic heterogeneity suggests that either modifier genes, epigenetic mechanisms or environmental factors could modulate the FAP phenotype. There is good evidence from humans, and particularly from mouse models, of the involvement of modifier genes that influence the severity of FAP. It is known that same sex siblings in their early twenties often show phenotypic differences which cannot be easily explained except by the action of modifier genes [30]. Despite those findings, further clinical information and an accurate follow-up of patients is necessary to confirm our results.

The two hotspots at codons 1061 and 1309 of the *APC *gene accounted in this study for 9.4% of the *APC*-positive families. However, they were reported mainly in Catalonian families. Mutation at codon 1061 was detected in 3 (CAT-03, CAT-04 and CAT-05) out of 13 Catalonian positive families (23%), but was not found in 19 *APC*-positiveGalician families. A similar trend was observed for the deletion at codon 1309: 15% (2 out of 13, CAT-10 and CAT-11) for Catalonians *vs *5,2% (1 out of 19, GAL-24) for Galician patients. Therefore, we performed statistical analyses in order to test whether the Galician population had a significantly different mutation frequency at these codons. Ji-squared tests yielded significance for the 1061 mutation (p = 0,058) but not for the 1309 variant (p = 0,356).

Although different mutation screening methods were used to study these two populations, the possibility that this fact could have caused the different spectra observed would be very small. Firstly, the mutation frequency in both populations was similar (40% for Galicia and 38% for Catalonia, p = 0.888). Besides, Galician samples were directly sequenced, which was the most sensitive of the techniques used.

Similar results had already been observed in a previous study where 15 unrelated Galician patients were analysed, but in that study it was not possible to establish whether the inability to detect the recurrent mutations at codons 1061 and 1309 actually reflected an underrepresentation of these genetic variations in this population, or was simply due to a sampling bias [10]. Furthermore, when we considered all the data available from the Galician FAP patients altogether (the 19 *APC*-positive families from this study plus the 6 from our previous one [10]), the frequencies observed were 4% (1/25 *APC*-positive) for the 1309 deletion, and still 0% for the deletion at codon 1061, which is certainly quite remarkable. Thus, we recalculated the Ji-squared test for the 1309 mutation and a trend towards the underrepresentation of this variant was indeed observed (p = 0,265).

Although there is plenty of evidence that the mutational spectrum of the *APC *gene varies in different populations, these two hotspots are thoroughly reported worldwide. They represent around 8% and 20% of the *APC*-positive families, and the 5 bp deletion at codon 1309 is reported as the most common germline mutation [31,32]. Interestingly, a frequency range for this deletion in different countries has also been described: a high rate in Japan (14%), a moderate frequency in most European populations (5–6%), and no 1309 deletions detected in Australia (0% out of 27 *APC*-positive families) [33]. It has also been recently reported that the frequency of the deletion at codon 1061 in 46 Czech and Slovak *APC*-positive families was lower than the expected (3%) [34]. The variation in the distribution of these hotspots could presumably be caused by an ascertainment bias, but in isolated populations it could as well be explained by a founder effect. For instance, in the Balearic Islands, where the hotspot at codon 1061 is overrepresented (50%), the haplotype analysis of the families sharing this deletion was consistent with the presence of a founder effect [35]. The recurrence of these two mutations has been linked to the molecular properties of the DNA region around codons 1061 and 1309, rather than with specific haplotypes. These two are located within a short hypermutable polyA repeat that may associate with an increased probability of DNA polymerase slippage during DNA replication, leading to an overrepresentation of deletions. The high incidence of the 1309 deletion among *de novo *cases, and the fact that this alteration was found to segregate with different haplotypes associated with the disease supports this hypothesis [33].

It is known that gene diversity in the Galician population is generally lower than in other European populations, as a result of its relative isolation from the rest of the Iberian Peninsula and the high emigration rates during the last two centuries [36,37]. These genetic features would have possibly caused the selection of not yet identified allelic variants in DNA repair genes. Hypothetically, those variants would repair more efficiently the DNA polymerase slippage caused by the repetitive sequences around codons 1061 and 1309 during replication. Therefore, lower frequencies for these two *APC *hotspots should be observed. Such founder effects have already been observed in this population for some genetic diseases, including *BRCA1 *in familial breast cancer [38].

### *MUTYH *mutations

Biallelic germline mutations in *MUTYH *were found in 24% of the *APC*-negative patients, i.e., 14/59 (fifty from this study plus 9 from the previous one [10]). This data is consistent with previous results [39,40]. Differences between these populations were not significant (p = 0.517).

Table 2 shows that the two most frequent mutations reported to date (p.Tyr165Cys and p.Gly382Asp) were detected in quite a number of cases, the frequency of these alleles being 71%. This observation is comparable to what has been described in the literature [9,41]. Among the other mutations found, the c.1186_1187*ins*GG accounted for 21% of the mutant alleles reported. This mutation was previously reported in Portuguese families with a similar frequency [22].

Biallelic *MUTYH *carriers displayed an attenuated polyposis phenotype without extracolonic manifestations, with the exception of patient CAT-19 who showed breast cancer at 59 years and CRC at 69 (Table 2). It is noteworthy that the BRCA1 and BRCA2 tumor suppressor proteins participate in the base excision repair of 8-oxo-7,8-dihydroguanine (8-oxoG) lesions [42]. Accordingly, loss of BER function due to biallelic *MUTYH *mutations may underlie breast cancer risk. In Dutch MAP patients, breast cancer occurred in 18% of females, significantly more than the expected from national statistics. This observed increased breast cancer risk should be thoroughly investigated [21].

The median age at diagnosis of CRC in MAP families was 51,5 years (ranging from 43 to 69) (Table 2). In contrast, classical FAP patients showed a CRC onset 10 years earlier (median 41, ranging from 20 to 46) (Table 1). As previously reported [8], it appears that disease symptoms in MAP are not as severe as those observed in *APC- *driven FAP, and that they resemble an attenuated polyposis phenotype. However, patients GAL-08, CAT-15, GAL-03 and CAT-18 presented ambiguous clinical manifestations, with a display of CRC at around their forties, which is more likely a feature of FAP, but a number of polyps and an onset typical of the attenuated phenotype. Hence, we have thought it appropriate to classify them as AFAP. We realise that classification of such patients is difficult, since it is well-known there is a lack of agreement concerning the exact diagnostic criteria that should be used to classify attenuated polyposis [43].

As expected, most of the bilallelic *MUTYH *carriers were found in families with an autosomal recessive model of inheritance, or in cases with apparent sporadic presentation. However, we identified three patients (CAT-14, GAL-22 and CAT-16) with a family history of vertical transmission of CRC; similar results had already been described [44].

Biallelic *MUTYH *mutations have been consistently linked to higher CRC susceptibility. However, the risk for monoallelic *MUTYH *carriers remains controversial. Balaguer *et al*. [26] used a meta-analysis of published case-control studies and concluded that monoallelic *MUTYH *carriers were not at increased risk for CRC, although an effect of bordeline statistical significance was observed for p.Tyr165Cys. In the present study, monoallelic changes with predicted functional relevance (p.Tyr165Cys, p.Gly382Asp, p.Val232Phe) were found in 3/45 patients, and accounted for 6.7% of cases. Nevertheless, they were not included as positive within the overall data, even though the p.Val232Phe was recently shown to reduce glycosylase activity [45].

Five new VUS were identified: c.-56 G>C, c.39 C>T (p.Ala13Ala), c.269A>G (p.Tyr90Cys), c.508C>T (p.Arg170Trp) and c.762 G>A (p.Gln254Gln). We also found p.Arg412Cys, previously described by Aceto *et al. *[46] and predicted by Polyphen as "possibly damaging". Neither of these variants were found in healthy controls when genotyping 500 chromosomes. It is quite remarkable though, that two of these previously not reported variants (p.Tyr90Cys and p.Arg170Trp), predicted as "probably damaging", were both found in CAT-22. This patient displayed multiple adenomas (50–60) and CRC at 53 years, but had no family history of polyposis. However, further studies are necessary to assess if these two variants are indeed deleterious.

## Conclusion

Our mutation detection rate for the *APC *gene (39%) is consistent with previous reports. Using standard methods of mutation analysis, such as sequencing, 11% of the classical FAP patients would not have been detected, so analysis of large rearrangements of the *APC *gene is strongly recommended. A genotype-phenotype correlation was found for most of the *APC *identified mutations, although the inter-family phenotypic variability observed would suggest the existence of genetic and/or environmental modifiers.

Besides, our data regarding the incidence of the 1309 and 1061 deletions in *APC *could indicate that in Galician patients the frequency of these two hotspot mutations is underrepresented. In our study, codon 1061 proved to be significantly different from the Catalonian and other Caucasian populations. We believe this might be due to the genetic isolation of the Galician population.

Biallelic germline mutations in *MUTYH *accounted for 24% of the families analysed, all of which displayed an attenuated polyposis phenotype and a CRC onset 10 years later than FAP. It was observed that a family history of vertical transmission of CRC did not rule out the possibility of biallelic *MUTYH *mutations.

In short, the overall results resemble those previously published and confirm that large rearrangements represent an important percentage of *APC *germline mutations. The lower frequency observed for the two hotspots of *APC *in Galician families has probably lead to a higher heterogeneity of *APC *mutations in this population. *MUTYH *analysis is also recommended for all *APC-*negative families even if a recessive inheritance is not confirmed. From a molecular point of view, these findings altogether have important implications for the design of mutation detection strategies, especially in Galician FAP families.

## Competing interests

The authors declare that they have no competing interests.

## Authors' contributions

NGF carried out the molecular genetic analysis in the Galician samples, mainly the analysis of large rearrangements in *APC *and sequencing of *MUTYH*, and participated in the design of the study. SCB and MM performed the molecular genetic analysis in the Catalonian samples, participated in the design of the study, and helped to draft and revise the manuscript. CF carried out sequencing and analysis of *APC *in Galician samples and participated in the correction of the manuscript. JM and IM carried out the molecular genetic analysis in the Catalonian samples, mainly the analysis of large rearrangements in *APC*, FISH analysis of the *APC *gene and sequencing of *MUTYH*. ACas, FB and BG gathered clinical information. A Cas, AV and ACar helped to draft and revise the manuscript. CRP conceived the study, participated in its design and coordination, and wrote the manuscript. All authors read and approved the final manuscript.

## Pre-publication history

The pre-publication history for this paper can be accessed here:



## Supplementary Material

Additional file 1 **Oligonucleotide sequences and PCR conditions used to amplify exons 1–14 of *APC***. Primer sequences and size of the amplification fragments were listed along with the PCR reaction and amplification conditions. Click here for file

Additional file 2 **Oligonucleotide sequences and PCR conditions used to amplify exons 1–16 of *MUTYH***. Primer sequences and size of the amplification fragments were listed along with the PCR reaction and amplification conditions. Click here for file
