# Supplementary material for: Molecular analysis of the APC and MUTYH genes in Galician and Catalonian FAP families: a different spectrum of mutations?
Source: BMC Med Genet. 2009 Jun 16;10:57. doi: 10.1186/1471-2350-10-57 (PMC2702373; doi:10.1186/1471-2350-10-57)
Supplement: Additional file 1 — Oligonucleotide sequences and PCR conditions used to amplify exons 1–14 of APC. Primer sequences and size of the amplification fragments were listed along with the PCR reaction and amplification conditions. [file 1471-2350-10-57-S1.doc]

**Supplementary Table 1. Oligonucleotide sequences and PCR conditions used to amplify exons 1-14 of *APC*.** Amplification was performed in the 25µl amplification mixture containing 100-200ng DNA, 1.5mM MgCl_2_, 0.2mM of each dNTP, 0.5mM of each primer and 1.25U of Taq DNA polymerase.

| Exon | Primer | Sequence 5´→3´ | Size (bp) | Amplification conditions* |
| --- | --- | --- | --- | --- |
| **1** | F | AACCTTATAGGTCCAAGGGTAG | 234bp | A |
|  | R | ACCTCAAGTTTACAAGAGGGAA |  |  |
| **2** | F | AAATACAGAATCATGTCTTGAAGT | 212bp | B |
|  | R | ACACCTAAAGATGACAATTTGAG |  |  |
| **3** | F | GACCCAAGTGGACTTTTCAGG | 423bp | B |
|  | R | ACAATAAACTGGAGTACACAAGG |  |  |
| **4** | F | GAGAAGTTTGCAATAACAACTGATG | 291bp | A |
|  | R | TTATCCTGAATTTTAATGGATTACCT |  |  |
| **5** | F | AACCTCACTCTAACTGGACCAA | 481bp | A |
|  | R | AACAGAGCTGTAATTCATTTTATTCC |  |  |
| **6** | F | GGTAGCCATAGTATGATTATTTCT | 204bp | B |
|  | R | CTACCTATTTTTATACCCACAAAC |  |  |
| **7** | F | AAGAAAGCCTACACCATTTTTGC | 238bp | B |
|  | R | GATCATTCTTAGAACCATCTTGC |  |  |
| **8** | F | GACACTTCATTTGGAGTACCTTAACA | 222bp | A |
|  | R | GGCATTAGTGACCAGGGTTT |  |  |
| **9** | F | AGTCGTAATTTTGTTTCTAAACTC | 394bp | B |
|  | R | TTTGAAACATGCACTACGAT |  |  |
| **10** | F | TTGCTCTTCAAATAACAAAGCAT | 192bp | A |
|  | R | TCCACCAGTAATTGTCTATGTCA |  |  |
| **11** | F | GATGATTGTCTTTTTCCTCTTGC | 215bp | B |
|  | R | CTGAGCTATCTTAAGAAATACATG |  |  |
| **12** | F | TGACAAAGGAAGAACAGATAGCA | 390bp | B |
|  | R | GCAGTGAGCTGAGATTGCAC |  |  |
| **13** | F | TTTCTATTCTTACTGCTAGCATT | 306bp | B |
|  | R | ATACACAGGTAAGAAATTAGGA |  |  |
| **14** | F | AGGGACGGGCAATAGGATAG | 390bp | A |
|  | R | GGTCTTTTTGAGAGTATGAATTCTG |  |  |

*A: an initial denaturation at 94ºC for 2min, followed by 40 cycles at 94ºC for 30", (50ºC, for exon 10, and 55ºC) for 45” and 72ºC for 1min, and a final extension step at 72ºC for 7min.

B: The touchdown PCR protocol consists on: an initial denaturation at 94ºC for 2 min, followed by 3 cycles (94ºC for 30s, 60ºC for 40s and 72ºC for 30s), 3 cycles (94ºC for 30s, 58ºC for 40s and 72ºC for 30s), 25 cycles (94ºC for 30s, 55ºC for 40s and 72ºC for 30s) and a final extension step at 72ºC for 7 min.
