# Supplementary material for: Molecular analysis of the APC and MUTYH genes in Galician and Catalonian FAP families: a different spectrum of mutations?
Source: BMC Med Genet. 2009 Jun 16;10:57. doi: 10.1186/1471-2350-10-57 (PMC2702373; doi:10.1186/1471-2350-10-57)
Supplement: Additional file 2 — Oligonucleotide sequences and PCR conditions used to amplify exons 1–16 of MUTYH. Primer sequences and size of the amplification fragments were listed along with the PCR reaction and amplification conditions. [file 1471-2350-10-57-S2.doc]

**Supplementary Table 2. Oligonucleotide sequences and PCR conditions used to amplify exons 1-16 of *MUTYH*.** Amplification was performed in the 25µl amplification mixture containing 100-200ng DNA, 1.5mM MgCl_2_, 0,2mM of each dNTP, 0.5mM of each primer and 1.25Uof Taq DNA polymerase. For long PCR, the reaction conditions were the same, except for:1.75mM MgCl_2_, 2.5mM of each dNTP, and 1.8U of Taq DNA polymerase.

| **Exon** | **Primer** | **Sequence 5´→3´** | Size (bp) | Amplification conditions* |
| --- | --- | --- | --- | --- |
| 1 | R | GACGTCTGAACGGAAGTTCG | 234bp | C |
|  | F | AGAGCGCAGAGGCTTTGA |  |  |
| 2 | R | CCTTCCCAGCCTGAATCTG | 250bp | C |
|  | F | CCTTGGAAGGCCTCAAAAT |  |  |
| 3-7 | R | GTTAGTTGGGGAAGCCCTA | 996bp | D |
|  | F | CACCTGATTGGAGTGCCAAGA |  |  |
| 6-10 | R | AGAGGCACAGGGTTGAGTGT | 948bp | D |
|  | F | TTGGGGTGGGTGTAGAGAAG |  |  |
| 10-14 | R | CAACAGAGCGATTCTCCGTCTC | 1276bp | D |
|  | F | GCTTCACAGCAGTGTTCCCT |  |  |
| 15 | *R* | AGTGAAGCCTGGAGTGGAGA | 289bp | C |
|  | F | TGAAGTTAAGGGCAGAACACC |  |  |
| 16 | R | CGAAACCAGTCTGAGCAACA | 397bp | C |
|  | F | GGGAAAGGGAGAGGACAA |  |  |

*C: an initial denaturation at 94ºC for 2min, followed by 40 cycles at 94ºC for 30", 60ºC for 30” and 72ºC for 1min, and a final extension step at 72ºC for 7min.

D: Long PCR, to amplify 3 overlapping fragments, consists on: an initial denaturation at 94ºC for 3 min, followed by 10 cycles (94ºC for 30s, 55 ºC for 40s and 68ºC for 4 min) and 20 cycles (94ºC for 30s, 55ºC for 40s and 68ºC for 4 min (with an increased rate of 20s per cycle), and a final extension step at 72ºC for 7 min.
